# Resveratrol inhibits angiotensin II-induced ERK1/2 activation by downregulating quinone reductase 2 in rat vascular smooth muscle cells

**DOI:** 10.1016/S1674-8301(12)60019-0

**Published:** 2012-03

**Authors:** Xiwen Zhang, Yao Wang, Weiwei Yang, Xiaofeng Hou, Jiangang Zou, Kejiang Cao

**Affiliations:** a Department of Cardiology, The First Affiliated Hospital of Nanjing Medical University, Nanjing, Jiangsu 210029, China;; b Department of Nephrology, Huai'an First People's Hospital affiliated to Nanjing Medical University, Huai'an, China

**Keywords:** resveratrol, quinone reductase 2 (NQO2), reactive oxygen species, extracellular signal-regulated kinase (ERK), vascular smooth muscle cells

## Abstract

Our previous studies showed that resveratrol could inhibit the proliferation of vascular smooth muscle cells (VSMCs) and repress mRNA and protein expression of quinone reductase 2 (NQO2). This study further explored the potential mechanisms whereby resveratrol inhibits the proliferation of rat VSMCs. Lentiviral vectors that incorporated *NQO2* small interfering RNA (siRNA) were constructed and transduced into rat VSMCs. The cell proliferation was detected using the bromodeoxyuridine (BrdU) assay. Cultured rat VSMCs were stimulated with angiotensin II and the level of reactive oxygen species (ROS) was measured using a ROS assay kit. A realtime quantitative PCR was used to detect *NQO2* mRNA levels. Extracellular signal-regulated kinase (ERK1/2) and NQO2 protein expression were determined by Western blotting analysis. The inhibitory effect of resveratrol (10 and 50 µmol/L) on the proliferation of rat VSMCs in the NQO2 siRNA group was significantly weaker than that in the normal and scrambled siRNA group (*P* < 0.01). The ROS level in the NQO2 siRNA and resveratrol (50 µmol/L) treatment groups were lower than that in the normal and scrambled siRNA groups (*P* < 0.01 in both). Compared with the normal and scrambled siRNA group, the phosphorylation of ERK1/2 was significantly decreased in the NQO2 siRNA and resveratrol (50 µmol/L) treatment group (*P* < 0.01 in both). In conclusion, high concentration of resveratrol inhibits angiotensin II-induced ERK1/2 phosphorylation and subsequent proliferation by down-regulation of NQO2 in cultured rat VSMCs.

## INTRODUCTION

Percutaneous coronary intervention (PCI) has been widely used in the treatment of coronary heart diseases[1]-[2], but the long-term overall efficacy of PCI may be compromised by the occurrence of restenosis[3]. Many clinical and experimental studies have demonstrated that the proliferation of vascular smooth muscle cells (VSMCs) plays a major role in the pathogenesis of restenosis[4]-[6]. Consequently, suppression of VSMC proliferation can be a useful therapeutic intervention for reducing the incidence of restenosis after PCI. Resveratrol, known to be abundantly present in wine, is thought to have cardiovascular protective effects and inhibit the proliferation of VSMCs.[7] Therefore, it is crucial to elucidate the mechanisms whereby resveratrol exerts its cardioprotective effects in VSMCs.

In 2004, Wang *et al*.[8] found that resveratrol may exert its diverse biological functions through interactions with quinone reductase 2 (NQO2), a resveratrol-targeting protein. Buryanovsky *et al*.[9] reported that resveratrol was a potent inhibitor of NQO2 activity *in vitro* and it specifically bound to the deep active-site cleft of NQO2. Our previous studies found that resveratrol could bind to and repress the mRNA and protein expressions of NQO2 in VSMCs[10]. However, the mechanism for the effect of resveratrol on the proliferation of VSMCs by NQO2 is unclear.

There is accumulating evidence that the renin-angiotensin system may contribute to the pathogenesis of chronic vascular disease. Angiotensin II is the most important contributing factor for the proliferation of VSMCs[11]-[12]. Previous studies showed that angio-tensin II could increase the activity of NAD(P)H oxidase, a major source of reactive oxygen species (ROS) in vascular tissues[13]. The augmented ROS level can activate extracellular signal-regulated kinase (ERK)1/2 and subsequent proliferation of VSMCs[14]-[15]. Although the mechanisms of the suppression of angiotensin II-induced VSMC proliferation by resveratrol have been partially elucidated[16]-[17], the intracellular mechanisms through repression of NQO2 remain unspecified. In the present study, we sought to investigate whether resveratrol inhibited angiotensin II-induced rat VSMC proliferation by inhibiting ERK1/2 phosphorylation through down-regulation of NQO2.

## MATERIALS AND METHODS

### Reagents, cells and animals

Angiotensin II was purchased from Sigma (St. Louis, MO, USA). Dulbecco's modified Eagle's medium (DMEM), fetal bovine serum (FBS), resveratrol, the Bromodeoxyuridine (BrdU) Cell Proliferation Assay Kit, specific short hairpin RNA (shRNA) for *NQO2* and negative control, reagents for cDNA synthesis and polymerase chain reaction (PCR), and the SYBR Premix Ex *Taq* (perfect real-time) Kit were obtained through companies previously described[10]. The ROS detection kit was purchased from Beyotime Co. (Jiangsu, China), and rabbit polyclonal antibodies for ERK1/2 and phospho-ERK1/2 were obtained from Cell Signaling Technology (Beverly, MA, USA). A goat polyclonal antibody for NQO2 was purchased from Santa Cruz Biotechnology (Santa Cruz, CA, USA).

The 293 cell line was purchased from the Cell Bank of Chinese Academy of Sciences (Shanghai, China). VSMCs were isolated from male Sprague-Dawley rat aortas, as previously detailed[10]. The explants were incubated in DMEM containing 20% FBS, 100 U/mL penicillin, and 100 µg/mL streptomycin at 37°C in a humidified atmosphere of 5% CO_2_. The medium was replaced every 3 d. Cells were sub-cultured once they reached 80%-90% confluence, and VSMCs from passages 3-8 were used for the experiments.

All experiments were performed in accordance with the protocols approved by the Institutional Animal Care and Use Committee and followed the Guide for the Care and Use of Laboratory Animals of Nanjing Medical University (China).

### Lentiviral vector construction, production, and transduction

The siRNA sequence was inserted into the pGCL-GFP lentiviral vectors between the *Hpa*I and *Xho*I sites upstream of the CMV-GFP gene. The siRNA expression cassette was under the control of a U6 promoter, and the lentiviral vectors bearing *NQO2* siRNA were termed Psc-NQO2. The negative control lentiviral vectors, termed Psc-NC, were also generated as described above.

The 293T cells were maintained in DMEM supplemented with 10% FBS. The medium (20 mL), containing 1.2×10^7^ cells, was seeded into 15 cm culture dishes. To generate lentiviral vectors, either 20 µg Psc-NQO2 or Psc-NC was transfected along with 15 µg pHelper 1.0 (*gag*/*pol* element) and 10 µg pHelper 2.0 (VSVG element) into 293T cells at 80% confluence using Lipofectamine 2000 (Invitrogen, Carlsbad, CA, USA) according to the manufacturer's instructions. The medium was replaced with fresh medium 8 h post transfection. The cell culture supernatant containing the vectors was collected 48 h after transfection. The vector supernatant was concentrated by ultracentrifugation at 4000 g for 10 min at 4°C and then titrated in 293T cells using fluorescence-activated cell sorting (FACS) analysis for green fluorescent protein (GFP) expression.

Rat VSMCs were seeded into 6-well culture plates 24 h prior to transfection at a density of 1×10^5^ cells/well. The cells were transduced with lentiviral vectors (5×10^9^ TU/mL, 2 µL per well) at a multiplicity infection (MOI) of 100 in the presence of 5 µg/mL polybrene for 12 h. After 24-h incubation, rat VSMCs were cultured in fresh DMEM for an additional 72 h. We then sorted out the cells from high fluorescent siRNA-transfected VSMCs before harvesting them to determine NQO2 expression.

### Cell proliferation assay

Resveratrol was added to the culture medium at a concentration of 0, 1, 10, and 50 µmol/L. VSMCs (2×10^5^ cell/mL) were seeded into 96-well culture plates (100 µL/well). The BrdU reagent was diluted (1:500) and incorporated into the proliferating cells during the final 24 h incubation. BrdU was detected using a mouse monoclonal antibody against BrdU (1:200). After the addition of goat anti-mouse IgG peroxidase-conjugated secondary antibody, substrate and stop solution, the amount of BrdU incorporated into the cells was measured as optical density (OD) using a spectrophotometer microplate reader at a wavelength of 450 nm.

### Detection of intracellular ROS

Intracellular ROS level was detected using an oxidation-sensitive fluorescent probe (DCFH-DA). The culture medium was replaced with fresh medium not containing FBS and incubated for 24 h. Rat VSMCs were incubated for 1 h in culture medium containing 10 µmol/L DCFH-DA to establish a stable intracellular level for the probe and then stimulated with 100 nmol/L angiotensin II for another 1 h. The cultured rat VSMCs were washed with phosphate buffered saline (PBS) three times. The DCF fluorescence intensity of the cells was detected using the FACSAria flow cytometer (Becton Dickinson, San Jose, CA, USA). For each sample, 10,000 events were collected.

### RNA isolation and real-time quantitative polymerase chain reaction (qRT-PCR)

Total RNA was isolated using TRIzol reagent (Invitrogen) according to the instructions by the manufacturer. Total RNA (1 µg) was reverse transcribed using an oligo(dT) 18 primer and AMV reverse transcriptase. A real-time quantitative polymerase chain reaction (qRT-PCR) was performed using the ABI Prism 7300 Sequence Detector System (Applied Biosystems, Foster City, CA, USA). The expression levels of NQO2 were analyzed with qRT-PCR using a SYBR Green dye. The primer sequences were as follows: rat glyceraldehyde phosphate dehydrogenase (GAPDH), 5′-TTCAACGGCACAGTCAAGG-3′ (forward) and 5′-CTCAGCACCAGCATCACC-3′ (reverse); rat NQO2, 5′-TGGGATAGAAGCCTATGAAGCCTAC-3′ (forward) and 5′-GGATTGCTGGAACGCTGAAC-3′ (reverse). The products were 369 and 134 bp in size, respectively, and relative mRNA abundance was calculated using the 2^−△△Ct^ method.

### Western blotting analysis

The culture medium was discarded and the six-well plate was washed three times with PBS. The plate was added with the lysis buffer containing PMSF and incubated on ice for 30 min. Then, the lysates were collected and clarified by centrifugation at 12000 rpm for 10 min at 4°C, and the supernatant was collected for protein analysis. The whole protein samples were boiled for 5 min in 5×loading buffer and separated using 10% SDS-PAGE. Proteins were transferred to PVDF membranes at a current of 300 mA for 90 min. The membranes were incubated in a blocking solution of 5% fat-free milk in Tris-buffered saline plus Tween 20 (TBST) for 1 h. Anti-GAPDH, NQO2 and ERK1/2 and phospho-ERK1/2 antibodies were used for the immunoblotting procedure and protein bands were visualized by ECL.

### Statistical analysis

The data were expressed as mean±SEM, and a statistical analysis was performed by one-way ANOVA. SNK method was used to perform multiple comparisons of treatment groups with the control group as ANOVA was significant. The results were considered statistically significant at *P* < 0.05.

## RESULTS

### NQO2 was downregulated in rat VSMCs by lentiviral vectors containing *NQO2* siRNA

**Fig. 1:**
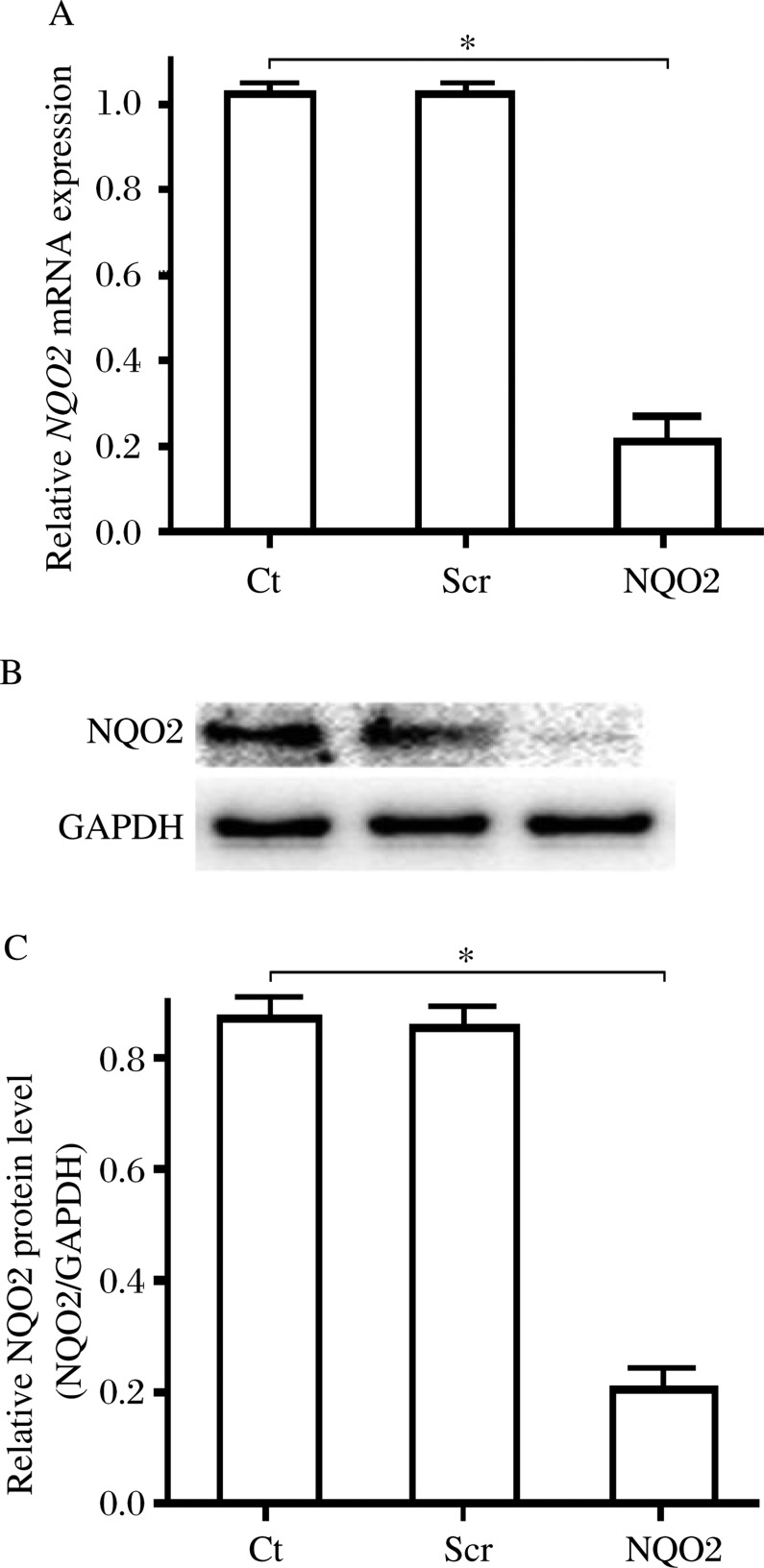
*NQO2* siRNA treatment inhibited the expression of NQO2. A: *NQO2* mRNA expression levels in the control, scrambled siRNA, and NQO2 siRNA groups using real-time quantitative PCR. B: Western blot analysis of NQO2 expression levels in the control, scrambled siRNA and NQO2 siRNA groups. C: Protein densitometry was performed and expression NQO2 was normalized against GADPH.Values are expressed as mean±SEM (*n* = 3). *P < 0.01. Ct: control; Scr: scrambled siRNA; NQO2: NQO2 siRNA.

The expression of NQO2 in lentiviral vectors-transduced VSMCs was evaluated by qRT-PCR and Western blotting analysis. qRT-PCR showed that *NQO2* siRNA significantly reduced the *NQO2* mRNA transcript levels by 79.1% at 72 h post transfection compared with the control group (*P* < 0.01, ***Fig. 1A***). The NQO2 protein level decreased by 76.4% in the NQO2 siRNA group 14 d after transfection compared with the control group (*P* < 0.01, ***Fig. 1B*** and ***1C***). There was no statistical difference between the scrambled siRNA and control group.

### NQO2 siRNA treatment attenuated inhibition by resveratrol of angiotensin II-induced proliferation of rat VSMCs

**Fig. 2:**
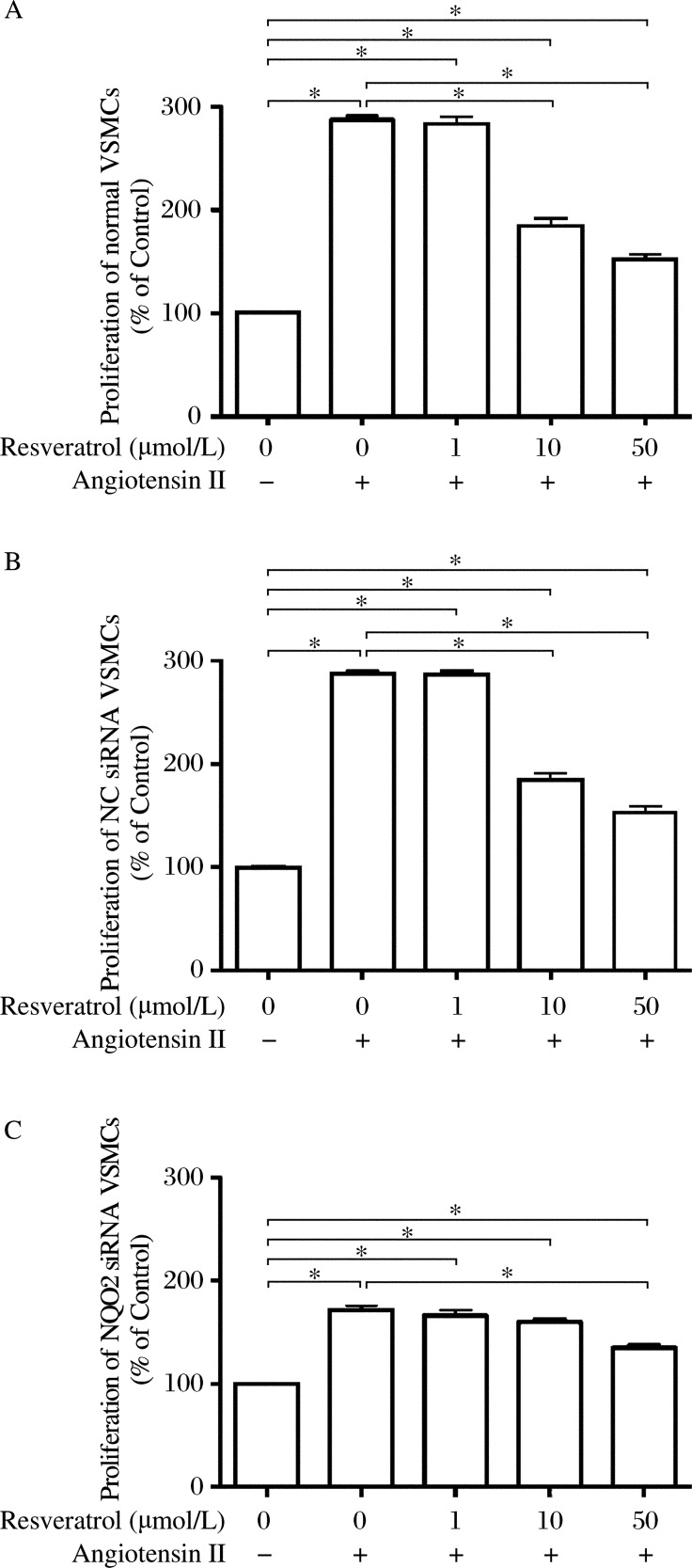
Resveratrol inhibits cell proliferation in the control (A), scrambled siRNA (B) and *NQO2* siRNA (C) treatment groups at 48 h. Cell proliferation was determined by the bromodeoxyuridine (BrdU) cell proliferation assay as described in “MATERIALS AND METHODS”. *NQO2* siRNA treatment attenuated the inhibition by resveratrol. Scrambled siRNA treatment had no effect on the proliferation of rat vascular smooth muscle cells (VSMCs). Values are expressed as mean±SEM (*n* = 6). ^*^P < 0.01.

After incubation in serum-free medium for 24 h, rat VSMCs were treated with 100 nmol/L angiotensin II for 48 h. Resveratrol was then added to the culture medium at 0, 1, 10, and 50 µmol/L. The proliferation of rat VSMCs in the three groups was inhibited by resveratrol in a concentration-dependent manner (***Fig. 2***). However, the inhibitory effect of resveratrol on the proliferation in the NQO2 siRNA group was significantly weaker than that in the other two groups. Resveratrol at 10 µmol/L effectively inhibited cellular proliferation in the control and scrambled siRNA group, respectively (*P* < 0.01 in both, ***Fig. 2A*** and ***2B***), but failed to noticeably inhibit cellular proliferation in the NQO2 siRNA group (*P* > 0.05, ***Fig. 2C***). Moreover, resveratrol at 50 µmol/L only reduced cellular proliferation by 20.98% in the NQO2 siRNA group; this was compared to a 46.78% reduction in the normal group and a 46.63% reduction in the scrambled siRNA group (*P* < 0.01 in both, ***Fig. 2***). There was no morphological evidence of any gross damage to rat VSMCs exposed to 1, 10, or 50 µmol/L resveratrol, respectively. These results strongly suggest that NQO2 siRNA significantly attenuates the inhibitory effect of resveratrol on cellular proliferation. In other words, resveratrol may inhibit rat VSMC proliferation *via* NQO2 suppression.

### Downregulation of NQO2 inhibits angiotensin II-induced generation of ROS in rat VSMCs

**Fig. 3:**
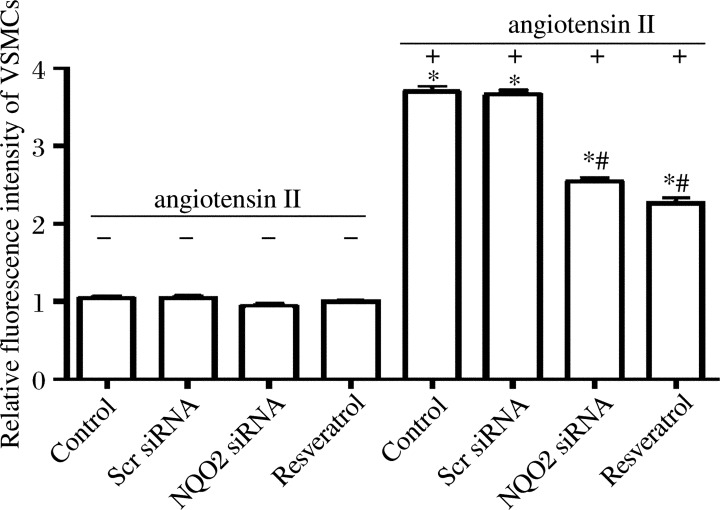
NQO2 siRNA and 50 µmol/L resveratrol inhibit angiotensin II-induced Intracellular ROS level was detected using an oxidation-sensitive fluorescent probe as described in Materials and Methods. intracellular ROS production in cultured rat vascular smooth muscle cells (VSMCs). Values are expressed as mean±SEM (*n* = 3). *P < 0.01 *vs* the control group in the absence of angiotensin II for 1 h (control); ^#^*P* < 0.01 *vs* the control group incubated with 100 nmol/L angiotensin II for 1 h. Scr: scrambled;

The effect of NQO2 downregulation on angiotensin II-induced generation of ROS was examined in cultured rat VSMCs. The lentiviral vectors without GFP were used to transfect rat VSMCs. After 24-h serum starvation, rat VSMCs were incubated with 100 nmol/L angiotensin II for 1 h. Prior to angiotensin II treatment, normal VSMCs were incubated with 50 µmol/L resveratrol for 30 min in the resveratrol treatment group. No statistical differences were observed when the cells were treated in the absence of angiotensin II among four groups. One h after treatment with 100 nmol/L angiotensin II, the ROS level in the NQO2 siRNA group was significantly lower than that in the control and scrambled siRNA groups (*P* < 0.01 in both). Scrambled siRNA had no inhibitory effect on ROS level when compared with control rat VSMCs (*P* > 0.05). Resveratrol at 50 µmol/L had a similar effect on ROS level to NQO2 siRNA (***Fig. 3***).

### Down-regulation of NQO2 inhibits angio-tensin II-induced activation of ERK1/2 in rat VSMCs

**Fig. 4:**
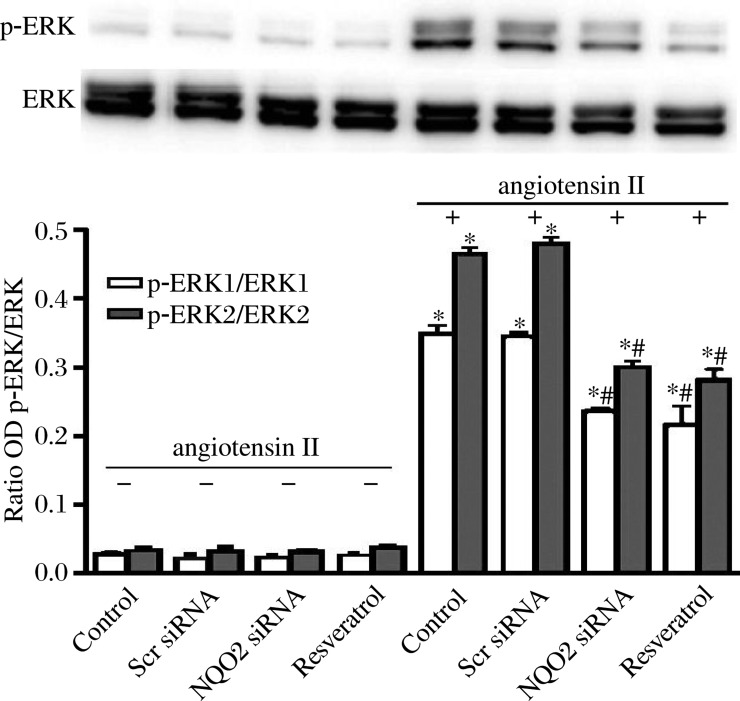
NQO2 siRNA and 50 µmol/L resveratrol inhibited angiotensin II-induced phosphorylation of ERK in cultured rat vascular smooth muscle cells (VSMCs). Phosphorylated ERK1 and ERK2 in VMSCs were examined by immunoblotting assays using phospho-specific antibodies against p-ERk1 and p-ERK2 and protein densitometry was performed. The expression of p-ERK1 and p-ERK2 was normalized against ERK1 and ERK2, respectively. Values are expressed as mean±SEM (*n* = 3). *P < 0.01 *vs* the control group in the absence of angiotensin II (control, C); ^#^*P* < 0.01 *vs* the control group incubated with 100 nmol/L angiotensin II for 10 min. Scr: scrambled.

Rat VSMCs were serum starved for 24 h and then treated with 100 nmol/L angiotensin II for 10 min or more, and harvested for Western blotting analysis. Prior to angiotensin II treatment, VSMCs were incubated with 50 µmol/L resveratrol for 30 min in the resveratrol treatment group. No statistical differences were observed among the four groups when the cells were treated in the absence of angiotensin II. In the presence of 100 nmol/L angiotensin II for 10 min, the phosphorylation of ERK was significantly decreased in the NQO2 siRNA group, compared with the control and scrambled siRNA group (*P* < 0.01 in both). Scrambled siRNA had no inhibitory effect on the phosphorylation of ERK, compared with control VSMCs (*P* > 0.05). Similarly, resveratrol, at 50 µmol/L, could repress ERK phosphorylation in the resveratrol treatment group (***Fig. 4***).

## DISCUSSION

In this study, we showed that resveratrol inhibited angiotensin II-induced proliferation of rat VSMCs by inhibiting ERK1/2 phosphorylation via the downregulation of NQO2. Growing evidence indicates that resveratrol is a natural antioxidant agent[18]-[19]. Pretreatment with resveratrol can lead to a marked reduction in intracellular accumulation of ROS through the upregulation of endogenous antioxidants and phase 2 enzymes in cultured VSMCs. Surprisingly, as a phase 2 enzyme, NQO2 expression is not upregulated by resveratrol[20]. Our previous studies showed that resveratrol can repress the expression of NQO2[10]. Consequently, NQO2 may have biological functions that are different from other phase 2 enzymes in VSMCs. In this study, we found that downregulation of NQO2 could inhibit angiotensin II-induced generation of ROS. ROS plays a critical role in the development of vasculopathies, including atherosclerosis, hypertension and restenosis after angioplasty[21]-[22]. ROS at low levels can act as secondary messengers, modulating the function of biochemical pathways and mediating responses such as growth of VSMCs. ROS at higher levels likely initiates the atherosclerotic events, resulting in an increased synthesis of numerous mitogenic factors that contribute to the hyper-proliferation of VSMCs[23]. Other studies also showed that the inhibition of NQO2 increased the concentration of endogenous electrophiles and consequently upregulated the expression of some antioxidant enzymes[9]. Gong *et al*.[24] found that NQO2 catalyzed the metabolic activation of vitamin K3, leading to cytotoxicity; however, NQO1 metabolically detoxifies vitamin K3 and protects cells against oxidative stress and other adverse effects space. These studies parallel our findings, suggesting that NQO2 may be involved in some adverse reactions in VSMCs.

NQO2 possesses a unique active-site cleft that can accommodate a trans-stilbene structure. It is shared among some natural polyphenols such as resveratrol, quercetin, apigenin, chalcone and some coumarin derivatives[9]. This research indicates that NQO2 may be a common target of the chemo-preventive polyphenols. Previous studies have only shown that NQO2 could combine with polyphenols in structure, but those studies have not specifically evaluated its function. We found that the inhibitory effect of resveratrol on the proliferation of NQO2 siRNA treated rat VSMCs was weaker than the control and scrambled siRNA treated rat VSMCs. This finding indicates that the downregulation of NQO2 undermined the inhibitory effect of resveratrol on VSMC proliferation, but the knockdown of NQO2 did not completely nullify the effect of resveratrol in NQO2 siRNA treated rat VSMCs. The two following reasons may explain this result: 1) There may be other resveratrol-targeting proteins in VSMCs that could combine with resveratrol. In 2008, Hsieh *et al*.[25] extracted glutathione sulfotransferase-pi (GSTP1), a new resveratrol-targeting protein, in cultured prostate cancer cells. 2) There still was a small number of normal rat VSMCs in the NQO2 siRNA group and resveratrol could inhibit their proliferation.

Increasing evidence has shown that ERK1/2 is a key enzyme involved in the regulation of cell proliferation[26]-[27]. Angiotensin II activates ERK1/2 and stimulates VSMC proliferation[28]-[30]. Our study found that the phosphorylation of ERK1/2 was significantly decreased in NQO2 siRNA treated rat VSMCs. For the inhibitory effect of resveratrol on angiotensin II-induced rat VSMC proliferation, one possible explanation may be that the suppression of NQO2 attenuates the formation of ROS and subsequently decreases the phosphorylation of ERK1/2. NQO2 is also required for TNF-induced activation of ERK1/2 in keratinocytes[31]. Accordingly, we speculate that NQO2 may have a pivotal role in different substance-induced cell signaling pathways.

Our research elucidates the potential intracellular molecular mechanism of resveratrol in the inhibition of rat VSMC proliferation. Through reducing NQO2 protein level and suppressing *NQO2* mRNA expression, resveratrol reduces ROS production and decreases the phosphorylation of ERK1/2 in cultured rat VSMCs. These findings show that NQO2 is an important target for resveratrol and has a potential therapeutic use for preventing the progression of cardiovascular disease.
